# Fertility status and depression: A case‐control study among women in Herat, Afghanistan

**DOI:** 10.1002/hsr2.70063

**Published:** 2024-09-09

**Authors:** Aziz‐ur‐Rahman Niazi, Mina Alekozay, Khadija Osmani, Abdul Fattah Najm

**Affiliations:** ^1^ Department of Public Health and Infectious Diseases, Faculty of Medicine Herat University Herat Afghanistan; ^2^ Mental Health Program, International Assistance Mission (IAM) Herat Office Herat Afghanistan; ^3^ Department of Surgery, Faculty of Medicine Herat University Herat Afghanistan

**Keywords:** Afghanistan, depression, Herat, infertility

## Abstract

**Background and Aim:**

On a global scale, an estimated 17.5% of the reproductive‐aged population experiences clinical infertility. Beyond its categorization as a reproductive health concern, infertility emerges as a substantial independent risk factor for the development of various mental health disorders. The current study aims to investigate the prevalence of depression among women experiencing infertility compared to fertile women in Herat city, Afghanistan.

**Methods:**

This hospital‐based case‐control study was conducted in Herat city, Afghanistan, during the latter half of 2023 on women aged 15–49. Sociodemographic and infertility data were gathered by a gynecologist using a structured questionnaire with 14 items. Assessment of depression employed the Dari‐translated version of the Patient Health Questionnaire. The data were scored and categorized following the instrument's guidelines. To examine the association between categorical variables, a chi‐square test was conducted, with a significance level set at 0.05 for all analyses. These statistical procedures were performed using IBM Statistical Package for Social Sciences, version 27.

**Results:**

Our investigation revealed a statistically significant association (*p* < 0.001) between infertility and depression. Infertility was associated with a markedly increased risk of depression, as evidenced by a prevalence of 79.0% in infertile women compared to 44.4% in fertile women. The study found a link between depression, health, and nutritional status in both infertile and fertile participants. Notably, within the infertile group, the longer a woman struggled with infertility, the more likely she was to experience depression.

**Conclusion:**

Given the substantial prevalence and important correlates of depression among infertile women in Herat, Afghanistan, it is imperative for policymakers, mental health professionals, and gynecologists to specifically address the mental well‐being of this vulnerable population.

## 1 INTRODUCTION

Infertility signifies the inability to achieve a pregnancy after a year of regular unprotected intercourse. This can stem from issues in either partner, highlighting the multifactorial nature of infertility.^1^ According to the World Health Organization (WHO), almost 17.5% of the global population experiences infertility, with a higher percentage in low and middle‐income countries (LMIC).^2^, ^3^

Beyond its classification as a reproductive health concern, infertility presents a significant risk factor for developing various mental health conditions.^3^, ^4^ Research suggests a relationship between infertility and mental disorders such as depression.^5^ Social stigma, fear of loneliness and divorce, traditional ideals, unknown causes of infertility, unpredictable treatment, and financial stress are critical factors for developing depression in infertile women.^6^, ^7^, ^8^, ^9^, ^10^ Psychological disorders in infertile women are followed by social isolation, low self‐esteem, and a low level of quality of life.^11^, ^12^

The prevalence of psychological disorders in women seeking infertility treatment is similar to that of patients with cancer.^13^ Globally, 44.32% and 28.03% of infertile women suffer from depression in LMIC and high‐income countries, respectively.^3^ It was estimated that the prevalence of depression among infertile women is 75.8% in Korea^14^ and Iran,^15^ 55% in Japan,^9^ 50% in Gaza,^16^ 48.4% in India,^17^ 31.3% in China,^4^ and 22% in Pakistan.^18^ Observed disparities in female infertility prevalence across diverse nations could potentially be attributed to variations in socioeconomic and cultural contexts.

Several studies have investigated the prevalence of depression within distinct demographic groups in Afghanistan. The findings indicate that depression affects approximately 4.8% of the Afghan population overall.^19^ These studies also reveal a concerning trend of elevated depression rates within specific subpopulations, with 69.6% of medical students and 42.8% of pregnant women in Herat city experiencing depression.^20^, ^21^

The scientific literature currently lacks research exploring the prevalence of depression amongst infertile women in Afghanistan. To address this gap in knowledge, the present study aims to assess the frequency of depression within this specific population residing in Herat city, Afghanistan.

## 2 METHODS

### 2.1 Study setting

This hospital‐based case‐control study was conducted in Herat, Afghanistan, during the latter half of 2023.

### 2.2 Target population

The target population of this study included women of reproductive age (14–49 years old) who resided in Herat city and visited the Osmani Obstetrics and Gynecology Clinic during the study period. According to Afghanistan National Statistics and Information Authority, Herat city was home to 631,883 people of whom 316,801 (50.1%) were women, and 151,012 (47.7%) were aged between 15 and 49 years.

### 2.3 Sample size and sampling procedure

A minimum sample size for the study was determined using the Raosoft sample size calculator. A 95% confidence interval with a 95% confidence level and an anticipated response distribution of 50% were employed. This resulted in a minimum recommended sample size of 384 participants. To account for potential issues of nonresponse, incomplete, or inaccurate data, the sample size was inflated by 10%, yielding a final sample of 422 participants. This comprised 211 participants with a diagnosed infertility condition and an additional 211 participants with normal reproductive function, matched on relevant demographic characteristics to the infertile group. The study employed a convenience sampling approach, where participants were selected based on their ease of access and suitability for the research question.

### 2.4 Data collection

An obstetrics and gynecology specialist gathered sociodemographic and infertility data using a structured questionnaire with 14 items. Questionnaires were administered orally. Each question and corresponding response options were read aloud to participants. Participants provided verbal responses, which were recorded manually on the questionnaire. Assessment of depression employed the Dari‐translated version of the Patient Health Questionnaire (PHQ‐9),^22^ with scoring and categorization adhering to the instrument's established guidelines. Briefly, Scores ranging from 0 to 4 indicated no or minimal depressive symptoms, 5 to 9 mild depression, 10 to 14 moderate depression, 15 to 19 moderately severe depression, and 20 to 27 severe depression. The PHQ‐9 instrument demonstrated high reliability in measuring depression symptoms for the sample population, as indicated by a Cronbach's alpha value of 0.841.

### 2.5 Statistical analyses

Statistical analyses were performed using IBM SPSS Statistics (version 26). Categorical data were expressed as frequencies and percentages, while continuous data were reported as medians and interquartile ranges. A binary depression variable was created. Participants with PHQ‐9 scores indicating mild, moderate, moderately severe, or severe depression were classified as depressed. Those with minimal or no depressive symptoms were classified as depression‐free. The relationship between categorical variables and infertility was assessed using a chi‐square test. Additionally, a Mann‐Whitney U test was employed to compare the median depression scores between the fertile and infertile groups. A *p*‐value of 0.05 was considered statistically significant for all analyses.

### 2.6 Ethical consideration

The study protocol received ethical approval from the Human Ethics Committee of Herat University (#230421). All participants were interviewed after providing written informed consent, with assurances of complete privacy and confidentiality. An obstetrics and gynecology specialist collected both research data and informed consent from infertile women after their second visit. To minimize potential coercion on patients, informed consent was obtained after initial assessments. The control group comprised fertile women related to the study participants.

## 3 RESULTS

### 3.1 Socio‐demographic data

The study included 415 women aged 14–49 years with a median age of 26.0 (22–30) years. Table 1 presents the sociodemographic characteristics of both the case and control groups. The median age at marriage for all participants was 19 (17–21) years. Of all participants, 288 (69.4%) were employed, 228 (54.9%) had an average family economy, 256 (61.7%) had a good nutritional status, 255 (61.4%) had a good health status, 136 (32.8%) had a bachelor degree, and 206 (49.6%) were infertile.

**Table 1:** Sociodemographic characteristics of participants.

Variables	Number	Percent (%)
**Age**		
14–25	207	49.9
26–35	170	41.0
>35	38	9.2
**Age at marriage**		
<15	50	12.0
15–25	340	81.9
26–35	24	5.8
**Job**		
Employed	288	69.4
Unemployed	127	30.6
**Family economy** *		
Good	132	31.8
Average	228	54.9
Poor	55	13.3
**Nutrition status** *		
Good	256	61.7
Average	134	32.3
Poor	25	6.0
**Health status** *		
Good	255	61.4
Average	127	30.6
Poor	33	8.0
**Education**		
Illiterate	86	20.7
Primary	86	20.7
High school	99	23.9
Bachelor	136	32.8
Master	8	1.9
**Infertility**		
Infertile women	206	49.6
Normal women	209	50.4

* Self‐perceived.

### 3.2 Prevalence of depression

Table 2 compares depression prevalence between the study and control groups. A statistically significant association (*p* < 0.001) was observed between infertility and depression, with infertile women exhibiting a higher prevalence rate. The prevalence of depression was notably higher in the infertile population, at 79.0%, compared to 44.4% in fertile women. The Mann‐Whitney U test revealed a significant difference in the level and severity of depression in the two study groups (*p* < 0.001).

**Table 2:** Association between depression and infertility.

	Infertile women	Fertile women
None‐minimal depression	43 (21.0%)	115 (55.6%)
Mild depression	71 (34.6%)	49 (23.7%)
Moderate depression	56 (27.3%)	34 (16.4%)
Moderate to severe depression	20 (9.8%)	8 (3.9%)
Severe depression	15 (7.3%)	1 (0.5%)
*p* value	<0.001	

### 3.3 Correlates of depression among study participants

Analysis of sociodemographic data revealed a significant association between depression with health and nutritional status across both study and control groups, as presented in Table 3. Specifically, within the infertile group, the duration of infertility emerged as a significant factor associated with depression prevalence (Table 4).

**Table 3:** Association between sociodemographic characteristics with depression in fertile and infertile women.

Variables	Infertile women			Fertile women		
	Depressed*	Normal*	*p* value	Depressed*	Normal*	*p* value
**Age**			0.390			0.873
14–25	79 (76.7%)	24 (23.3%)		45 (47.9%)	59 (51.3%)	
26–35	71 (83.5%)	14 (16.5%)		40 (42.6%)	45 (39.1%)	
>35	13 (72.2%)	5 (27.8%)		9 (9.6%)	11 (9.6)	
**Age at marriage**			0.150			0.904
<15	28 (87.5%)	4 (12.5%)		9 (9.6%)	9 (7.8%)	
15–25	123 (78.8%)	35 (22.2%)		81 (86.2%)	101 (87.7%)	
26–35	12 (80.0%)	3 (20.0%)		4 (4.3%)	5 (4.3%)	
**Job**			0.143			0.893
Employed	137 (81.1%)	32 (18.9%)		54 (45.4%)	65 (54.6%)	
Unemployed	26 (70.3%)	11 (29.7%)		40 (44.4%)	50 (55.6%)	
**Family economy**			0.110			0.155
Good	35 (70.0%)	15 (30%)		33 (40.2%)	49 (59.8%)	
Average	87 (79.8%)	22 (20.2%)		55 (46.2%)	64 (53.8%)	
Poor	41 (87.2%)	6 (12.8%)		4 (66.7%)	2 (33.3%)	
**Health status**			0.001			0.002
Good	73 (69.5%)	32 (30.5%)		56 (37.3%)	94 (62.7%)	
Average	64 (86.5%)	10 (13.5%)		34 (64.2%)	19 (35.8%)	
Poor	26 (96.3%)	1 (3.7%)		4 (4.3%)	2 (1.7%)	
**Nutrition status**			0.001			0.006
Good	86 (71.1%)	35 (28.9%)		50 (37.0%)	85 (63.0%)	
Average	56 (87.5%)	8 (12.5%)		41 (58.6%)	29 (41.4%)	
Poor	21 (100%)	0 (0.0%)		3 (75.0%)	1 (25.0%)	
**Education**			0.656			0.383
Illiterate	56 (84.8%)	10 (15.2%)		7 (35.0%)	13 (65.0%)	
Primary	40 (75.5%)	13 (24.5%)		19 (57.6%)	14 (42.4%)	
High school	36 (2.1%)	10 (23.3%)		25 (47.2%)	28 (52.8%)	
Bachelor	30 (75.0%)	10 (25.0%)		39 (40.6%)	57 (59.4%)	
Master	1 (100)	0		4 (57.1%)	3 (42.9%)	

* A binary depression variable was created. Participants with PHQ‐9 scores indicating mild, moderate, moderately severe, or severe depression were classified as depressed. Those with minimal or no depressive symptoms were classified as depression‐free.

**Table 4:** Association between infertility characteristics with depression among infertile women.

Variables	Responses	Depressed *N* (%)	Normal *N* (%)	*p* value
**Type of infertility**				0.929
Primary	121	96 (79.3)	25 (20.7)	
Secondary	85	67 (78.8)	18 (21.2)	
**Cause of infertility**				0.515
Women related factors	141	114 (80.9)	27 (19.1)	
Men related factors	11	9 (81.8)	2 (18.2)	
Both	13	11 (84.6)	2 (15.4)	
Unknown	41	29 (70.7)	12 (29.3)	
**Duration of infertility**				0.038
0–6 months	43	31 (72.1)	12 (27.9)	
7–12 months	40	27 (67.5)	13 (32.5)	
13–24 moths	17	13 (76.5)	4 (23.5)	
>24 months	106	92 (86.8)	14 (13.2)	
**Family support**				0.082
Yes	174	134 (77.0)	40 (23.0)	
No	32	29 (90.6)	3 (9.4)	
**Husband support**				0.286
Yes	188	147 (78.2)	41 (21.8)	
No	18	16 (88.9)	2 (11.1)	

## 4 DISCUSSION

This study aimed to compare the prevalence and associated factors of depression among fertile and infertile women in Herat city of Afghanistan. The prevalence of depression among infertile women was 79.0%. This is consistent with the results of a study in South Korea, which reported that 75.8% of participants suffered from definitive depression.^14^ On the other hand, the finding of this study was much higher than the results obtained from studies in Saudi Arabia,^23^ India,^17^ Iran,^3^ Vietnam,^24^ China,^4^ Gaza,^16^ Bangladesh,^25^ and Iraq.^26^ No other study found a depression prevalence higher than what we found in this study. This indicates that the prevalence of depression among infertile women in Herat is alarmingly high and it needs thorough evaluation and intervention.

Among the infertile women, the duration of infertility was significantly associated with depression. This finding is in line with a study in Iran,^3^ and Iraq,^26^ which also found a significant association between the duration of infertility and depression. On the other hand, other studies from Iran,^15^ Gaza,^16^ and the Republic of Korea^14^ revealed an insignificant association between the duration of infertility and depression among women. It can be argued that there is a relationship between depression and the length of infertility. This may be due to the fact that when infertility is diagnosed early, there is a greater sense of hope for successful treatment. However, as time passes and despite undergoing various therapies, the failure to conceive can lead to feelings of frustration and stress, ultimately contributing to depression in affected females.^27^, ^28^

This investigation found no significant association between the type of infertility and depression levels in infertile women. This aligns with previous studies from Turkey,^29^ and Pakistan,^18^ reporting similar null findings. However, discordant results emerged from studies conducted in Iraq,^26^ and Gaza,^16^ where a significant association between infertility type and depression was observed. Women from different cultural backgrounds may have different responses to infertility and the medical treatment involved. While women who have already had one child may feel less pressure to conceive and therefore be more comfortable with infertility issues, it is generally expected that those who are experiencing secondary infertility will be more at ease with the situation compared to those who have never had a child.^28^ These contrasting findings underscore the need for further research to elucidate the potential moderating or mediating factors influencing the relationship between infertility type and depression, considering regional and sociocultural differences.

Our analysis revealed no significant relationship between the cause of infertility and depression in infertile women. This mirrors the findings of South Korean^14^ and Iran studies.^6^ However, our results diverge from studies conducted in Vietnam,^24^ and Gaza,^16^ where a link between the cause of infertility and depression was identified. These discrepant observations necessitate further investigation to unravel potential factors, such as cultural context or specific diagnoses, that might influence this association.

One significant finding from this study was the correlation between the nutrition status of infertile women and their levels of depression. This finding is consistent with a Korean study that also showed a significant link between nutrition status and depression in infertile women.^14^ It was found that poor nutrition status in infertile women increased the likelihood of depressive symptoms compared to normal women. Additionally, our study found a significant correlation between health status and depression in infertile women, which is in alignment with the Korean study's findings.^14^ This highlights the importance of good nutrition and health status in preventing or decreasing the level of depression among women, especially those with infertility.

### 4.1 Limitation

The current investigation possesses limitations. Firstly, the ascertainment of depression solely relied on the PHQ‐9 instrument, while the established gold standard for depression diagnosis incorporates both physical examination and comprehensive clinical evaluation. Secondly, the sample population was restricted to patients referred to a private clinic in Herat, limiting generalizability to patients utilizing alternative healthcare facilities within the province and the country. Thirdly, given the high illiteracy rates in Afghanistan, self‐reported questionnaires were not feasible. The administration of interview‐based questionnaires raises concerns about potential response bias in our findings. Finally, a potential methodological limitation arises from the dual role of the obstetrics and gynecology specialist as both researcher and data collector. This arrangement could have introduced a risk of participant bias, as individuals may have felt compelled to participate due to concerns about potential repercussions for their care should they decline involvement in the study.

### 4.2 Recommendation

A more extensive study, involving clinical and physical assessments, is advised to investigate depression among infertile women. Furthermore, implementing interventions targeted towards enhancing mental health outcomes in infertile women may be warranted. Such interventions could encompass educational workshops, dissemination of informative materials through diverse media channels (e.g., mass media), readily accessible free counseling services, and public health campaigns focused on both novel infertility treatment technologies and the psychological sequelae associated with infertility. Furthermore, the development of educational programs by family therapists specifically designed to augment couples' knowledge and understanding regarding infertility and its potential psychological impact is recommended.

## 5 CONCLUSION

The study demonstrated a statistically‐significant elevation in depression scores within the infertile cases compared to the fertile control group. The significant association between depression and both case and control groups highlight the importance of these factors in reducing the level of depression in the community. Policymakers, mental health professionals, and gynecologists should pay specifical attention to address the mental well‐being of women in Afghanistan.

## AUTHOR CONTRIBUTIONS

**Aziz‐ur‐Rahman Niazi**: Conceptualization; investigation; writing—original draft; methodology; writing—review and editing; software; formal analysis; supervision; project administration; data curation. **Mina Alekozay**: Conceptualization; investigation; writing—original draft; methodology; writing—review and editing; formal analysis. **Khadija Osmani**: Conceptualization; investigation; writing—original draft; writing—review and editing; methodology. **Abdul Fattah Najm**: Conceptualization; investigation; funding acquisition; writing—original draft; writing—review and editing; methodology; software; formal analysis.

## CONFLICT OF INTEREST STATEMENT

The authors have declared that no competing interests exist.

## TRANSPARENCY STATEMENT

The lead author Aziz‐ur‐Rahman Niazi affirms that this manuscript is an honest, accurate, and transparent account of the study being reported; that no important aspects of the study have been omitted; and that any discrepancies from the study as planned (and, if relevant, registered) have been explained.

## Data Availability

The data that support the findings of this study are available from the corresponding author upon reasonable request.
